# Hardware for Recognition of Human Activities: A Review of Smart Home and AAL Related Technologies

**DOI:** 10.3390/s20154227

**Published:** 2020-07-29

**Authors:** Andres Sanchez-Comas, Kåre Synnes, Josef Hallberg

**Affiliations:** 1 Department of Productivity and Innovation, Universidad de la Costa, Barranquilla 080 002, Colombia; 2 Department of Computer Science, Electrical and Space Engineering, Luleå Tekniska Universitet, 971 87 Luleå, Sweden; josef.hallberg@ltu.se

**Keywords:** smart home, AAL, ambient assisted living, activity recognition, hardware, review

## Abstract

Activity recognition (AR) from an applied perspective of ambient assisted living (AAL) and smart homes (SH) has become a subject of great interest. Promising a better quality of life, AR applied in contexts such as health, security, and energy consumption can lead to solutions capable of reaching even the people most in need. This study was strongly motivated because levels of development, deployment, and technology of AR solutions transferred to society and industry are based on software development, but also depend on the hardware devices used. The current paper identifies contributions to hardware uses for activity recognition through a scientific literature review in the Web of Science (WoS) database. This work found four dominant groups of technologies used for AR in SH and AAL—smartphones, wearables, video, and electronic components—and two emerging technologies: Wi-Fi and assistive robots. Many of these technologies overlap across many research works. Through bibliometric networks analysis, the present review identified some gaps and new potential combinations of technologies for advances in this emerging worldwide field and their uses. The review also relates the use of these six technologies in health conditions, health care, emotion recognition, occupancy, mobility, posture recognition, localization, fall detection, and generic activity recognition applications. The above can serve as a road map that allows readers to execute approachable projects and deploy applications in different socioeconomic contexts, and the possibility to establish networks with the community involved in this topic. This analysis shows that the research field in activity recognition accepts that specific goals cannot be achieved using one single hardware technology, but can be using joint solutions, this paper shows how such technology works in this regard.

## 1. Introduction

Smart home (SH) technology moved in the last decade beyond a research field into a commercial enterprise. In the beginning, SH technology was applied strongly in security and surveillance, energy-saving, and entertainment, among others. Nowadays, the landscape has expanded with technologies such as the Internet of Things (IoT), artificial intelligence (AI), and computing techniques, helping to focus research and development (R&D) on working in fields such as improving the standard of living and autonomy for elder or disabled people, among others [1], this raise questions such as what can houses do for inhabitants’ needs, and how. A smart home can improve inhabitants’ lives when it is capable of sensing, anticipating, and responding to their daily activities, assisting them in a socially appropriate and timely way [2]. A basic smart home system is composed of an Internet connection, a smart home gateway, and devices connected as multiple nodes in the system [3], with nodes as sensors and actuators with wired or wireless communication [4]. This amount of data generation requires data processing techniques, allowing research areas such as ubiquitous and mobile computing to emerge as vital components of surveillance, security, and ambient assisted living, requiring research on human activity recognition. Some research fields have emerged as well, such as wearable sensor-based activity monitoring as a result of sensors deployed over the human body, and dense sensor-based activity monitoring from sensor network technologies, smart sensors, or smart appliances, among others [5].

The concept of “activity” itself, as what can be performed by a person, is the core for constructing applications or concepts like ambient assisted living (AAL) [6]. The complexity of the activity recognition problem increases with the complexity of the activity. Researchers are focusing on complex activity recognition, for example, using a computer, which involves other activities such as typing, using a mouse, sitting [7], etc., as well as those activities with longer duration composed of multiple actions and sequences of simple activities [8]. Thus arose the need to develop solutions around smart home concepts using hardware and software capable of capturing residents’ behavior and understanding their activities, informing them of risk situations, or taking action for their satisfaction [9]. Event recognition and emotion recognition are also part of this technology concept [10]. The smart home is considered as a technology that can help reduce the cost of living and care for the elderly and disabled population, and improve their quality of life. This concept is also applicable to solutions like energy saving, security management, and risk detection, such as fire, e.g., using such technologies as video monitors, alarms, planners and calendars, reminders, sensors, or actuators, among others [9]. All of the above complements the vision of Mark Weiser [11], allowing those research fields as pervasive or ubiquitous as computing bear vanguard systems such as AAL [12], which are context-aware, personalized to individual needs, adaptive to changing individual needs, ubiquitous in our everyday environment, and remain transparent in individual daily life [13]. The importance of developing these systems lies in their capacity to empower people’s activities through digital environments capable of sensing, adapting, and responding to human needs. In addition, these systems identify actions, habits, gestures, emotions, and establish a pervasive and unobtrusive human–machine communication interaction [13].

Both smart home and AAL needs for activity recognition developments are based on hardware and software capabilities. It is worth noting that activity recognition depends on data gathered from sensor systems, but the core is the data processing system based on software development. Therefore, trying new approaches, models, and algorithms with new data captured each time could be expensive. That is why activity recognition datasets freely accessible for R&D, gathered from research at specialized facilities by research institutes, helped to generate an explosion of knowledge in computer science around artificial intelligence problems, methods, tools, and techniques. A recent review of datasets for activity recognition is presented by [14]. CASAS (Center for Advanced Studies in Adaptive Systems) and UCI Human Activity Recognition dataset, among others, are the most popular for activity recognition system development, used by [15,16], respectively. Despite such advances in research, it is good to study all perspectives of the research context in AR to boost technological advances further in smart home and AAL. This review contributes by complementing the knowledge pool of software solutions with a broad overview of the hardware technology used for activity recognition applied in the field of smart homes and ambient assisted living. This work, covering the hardware technology used for AR, is not exhaustive but does give an extensive overview of recent technology in smart home and AAL. However, this paper focuses only on published studies in which researchers tested software development on hardware technology they used themselves, as this review seeks mainly to provide a road map for hardware solutions of activity recognition for smart home and AAL.

As activity recognition has been a growing research area in the last decade, while exploring the scientific literature retrieved from the search, we found several reviews related to hardware for smart home and AAL. Health is an interesting sector for these fields. Kötteritzsch [17] analyzed ambient assisted living systems in urban areas, focusing on assistive technologies for older adults, this work identified three categories to help classify AAL systems and pointed out challenges and future trends. Kötteritzsch [17] also found six hardware technologies proposed for use in AAL for older adults: wireless sensor network (WSN), camera, global positioning system (GPS), radiofrequency, and laser. Ni [18] presented a survey of elders independent living, characterizing the main activities considered in smart home scenarios, sensors, and data processing methods to facilitate service development. He offered some guidelines to help select sensors and processing techniques, grouping them into five categories for smart home environments for independent elders: environmental, wearable, inertial, and vital signs sensors. Acampora [13] discussed the emergence of AAL techniques in the health domain, examining body area networks (BANs) and dense/mesh sensor networks as infrastructure and sensor technologies in ambient sensor architecture. He summarized the hardware required for developing ambient intelligence systems based on special boards with Bluetooth and Zigbee for communication among the sensors, and sensors like accelerometer/gyroscope, blood glucose, blood pressure, electrocardiogram (ECG), electroencephalogram (EEG), electromyography (EMG), pulse oximetry, and humidity and temperature sensors. Expanding the study landscape, Kumari [6] presented a survey on increasing trends of wearables and multimodal interfaces for human activity recognition, discussing basic requirements, architectures, the current market situation, and developments using wearable sensors and bio-potential signals. Bejarano [1] reviewed the literature from 2010 to 2015 on technical and functional aspects of identifying common aspects such as architecture, network topology, scientometric information, and components of a smart home system, and described the uses, among other aspects. Peeton [19] investigated what kind of technologies exist to monitor activities at home for elderly people living independently, identifying five main groups of monitoring technologies: passive infrared (PIR) motion sensors, body-worn sensors, video monitors, pressure sensors, and sound sensors.

Although several review papers have been published over the years, considering the wealth of literature about applications, architectures, component functionality, and analysis comparing performance among the studies published in different sector applications, there are no broad studies related to the hardware used in activity recognition for smart home and AAL. This study is exploratory and has limitations; this paper does not study accuracy and performance, as they depend on more variety of data processing techniques, which are not the focus of the study. Nor was the level of acceptance, as many of these works were at a low development level, and many were laboratory tests. Even so, we do not limit the scope; we study and characterize the different uses or applications in which the hardware technology was used. We believe that this study provides an insightful overview of the hardware being used in AR, refreshes the knowledge in this area, and provides a different organization of the technology for smart home and AAL. This work is not a data summarization since bibliometrics networks allowed us to identify gaps in the new relationships between technologies, informing researchers and developers on current practices of how the available hardware is being used to develop useful applications on activity recognition for smart home and AAL. Besides, knowledge about what has not yet been tried can be retrieved, prompting valuable insights for novel development approaches and future research, promoting new combinations of ideas or uses of hardware technology through innovative strategies like the Medici effect [20], and contributing to possibly disruptive innovations. This research work can help future researchers to identify new systems based on the hardware being created in the AR field, map those developments, and strengthen their research. We also aimed to identify new research questions as input for new AR hardware development and highlight possible approaches that could potentially impact needs in the near future for smart home and AAL applications.

In this review, it is likely that, due to the broad and interdisciplinary nature of this applied technology and its research area, some relevant articles have been disregarded because they are not clearly identifiable in the titles or abstracts, or due to our inclusion and exclusion criteria or the choice of our key terms to build the query strings, also, due to the journal’s database selected. The review method is described in detail in Section 2. We present in Section 3, a brief scientometric and relational analysis of the research works chosen for the review, as well as the AR technology used in smart homes and AAL. The discussion in Section 4 points out interesting gaps in hardware technology combinations, and new potential studies around hardware technology for activity recognition are proposed. The review concludes in Section 5.

## 2. Review Method

This work, conducted as a systematic literature review, was executed in four stages, following PRISMA [21] guidelines, and the review approach for structuring the information was gathered from [22]. We applied software for visualizing bibliometric networks [23] in the first stage for the construction of query strings; the second stage focused on gathering potential results in the Web of Science (WoS) database; the third focused on excluding and including results based on criteria. Finally, the fourth stage consisted of characterizing the selected literature. The search was initially guided by wide concepts, but firmly focused on four technological areas of interest: smart home, smart environment, activity recognition, and ambient assisted living. The review did not consider gray literature.

Although WoS has many characteristics in common with Scopus in terms of indexed journals based on quality, they also differ, according to [24], in coverage and accuracy. We considered even though Scopus covers more journals than WoS, according to [25], Scopus tends to neglect indexing more papers, causing the loss of possible relevant works for our study. WoS has a stronger tendency to index journals in the science and technology field [26], as well as better accuracy in journal classification [24]. The above, in conjunction with the review method and the inclusion and exclusion criteria, helped to reduce the efforts of exploring quality scientific information, as the review seeks to capture a broad panorama of AR hardware technology with recent experimentation.

### 2.1. Query String Construction

**Figure 1:**
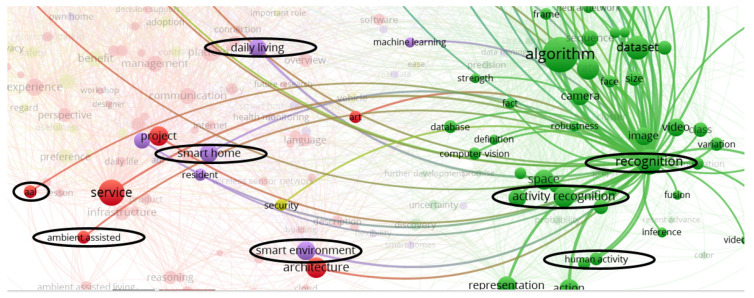
Some terms selected in network visualization of the bibliometric analysis generated in VOSviewer software.

Seeking to minimize the risk of overlooking relevant papers due to the choice of our key terms and to cover as many contributions as possible, a bibliometric networks analysis conducted in VOSviewer software [27] allowed us to get the best relevant terms used around the four areas of interest using titles, abstracts, and key terms. We retrieved from the WoS database the 100 most cited articles, and terms from all articles by the three most relevant authors as indicated by Google Scholar profiles from each area of interest: smart home (SH) and smart environment (SE), activity recognition (AR), and ambient assisted living (AAL). We generated different networks in VOSviewer to see the most mentioned words related to the more relevant terms, and to identify those that were semantically related and used once or a few times. This analysis helped select those terms that were synonymous with the areas of interest, common terms, strongly related terms, and synonyms, as shown in Figure 1.

**Figure 2:**
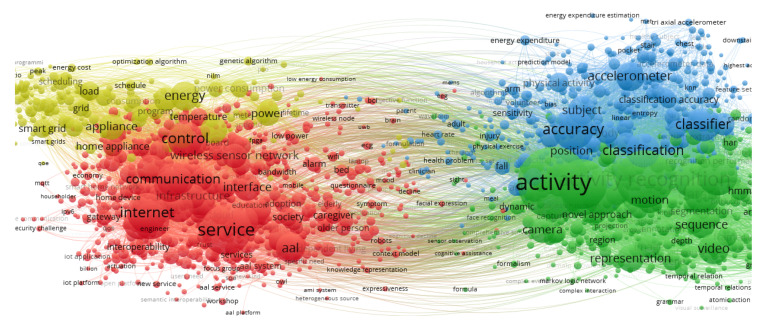
Biggest bibliometric network visualization of mixing papers retrieved from the World of Science (WoS) around the terms smart home, smart environment, activity recognition, and ambient assisted living.

Per area of interest (SH, SE, AR, AAL), we grouped and counted the selected terms to check duplication across the analysis, and chose common terms from concepts formed by one or more words. Finally, from the four terms (smart home, smart environment, activity recognition, ambient assisted living), we built three primary query strings (Table 1). Seeking to minimize the number of results per query and simplify the search, we supported the relationship of terms in the bigger bibliometric network visualization shown in Figure 2. As can be seen, AAL and smart home/environment are in the same cluster (red), and activity recognition is in a different cluster (green). Then, we combined the three primary query strings into two final query strings (FQ):

- FQ1: (AAL query) × (AR query)
- FQ2: (SH query) × (AR query)

### 2.2. Gathering Potential Results

**Figure 3:**
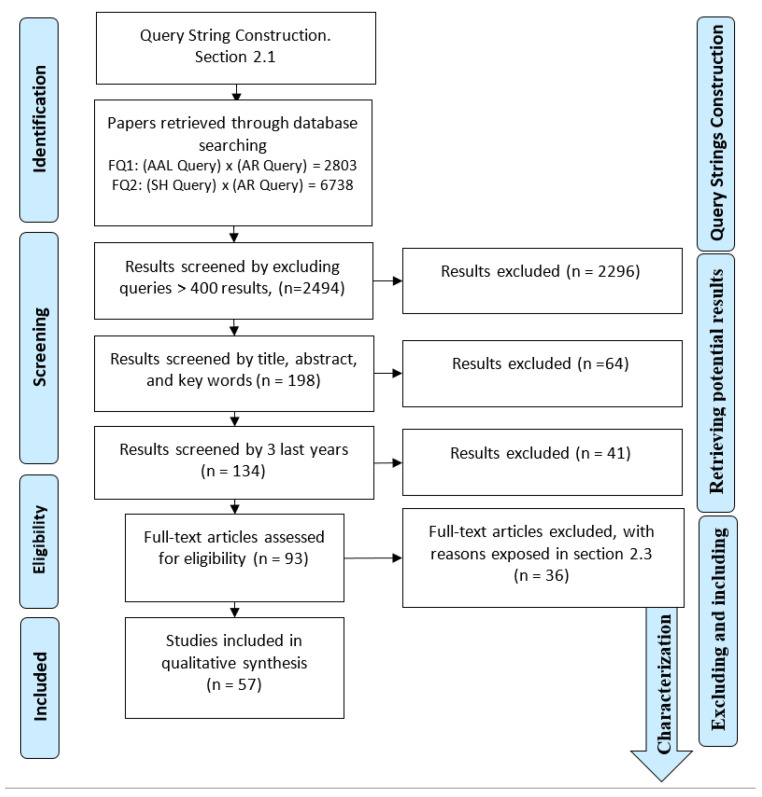
Diagram of the review method conducted based on PRISMA and operative structure in [28].

Testing FQ1 and FQ2 in WoS showed that the results were too big (Figure 3), so we decided to build 32 more reasonable small queries, from which we excluded queries with more than 400 results, considering them not reasonable to look at. For those with fewer than 400, based on the classification criteria used in [19], we checked the title and abstract shown in the results listed by the database as relevant or at least possibly relevant. For this, we used the match criterion “if it was about a technique or the use of technology and if the database was self-generated, but not acquired from a public one,” gathering 196 potential papers (Figure 3). As this amount was not suitable, we selected 2016, 2017, and 2018 as the last three years of the technology concept, obtaining 131 articles.

### 2.3. Including and EXCLUDING Results

In order to reduce even more the number of papers to be characterized, the aim at this stage was to get a final list, so for those papers still marked as dubious, we checked the whole paper to see whether it matched or not, looking for exclusions, using the following criteria:

- Proposal schemes and approaches, simulated scenarios or datasets, use of open or popular or well-known datasets, without proved experiment.
- Proposals of methodologies, approaches, frameworks or related that do not mention explicit testbeds, prototypes, or experimentation with hardware.
- Home automation applications, brain or gait activity recognition, health variables, or proposals to improve systems or cognitive activity.

As the focus of this work was to get the latest hardware technologies used in activity recognition research around smart homes and ALL, we considered the following criteria:

- The paper used hardware to acquire information for AR in the same research work.
- Datasets in the research work generated in the same experiment were used.
- Commercial technology, self-built devices, or developed prototypes were used.
- Tested approaches with self-built datasets using virtual or physical sensors on smartphones, smartwatches, smart bands, etc.
- There was a focus on testing and using hardware, acquired, or self-developed as part of the research.

As example, papers like “3D Printed ‘Earable’ Smart Devices for Real-Time Detection of Core Body Temperature” [29] were not included, because the main objective was only temperature detection, and not recognition of human activities.

### 2.4. Characterization of the Selected Literature

This final stage consisted of more profoundly analyzing the information and filling the technical characterization tables, which consisted of mainly gathering information about the hardware systems for activity recognition, their uses, the population or commercial target, the types of technologies, hardware references or models, and scientometric behaviors as guidance to establish research networking. We selected 56 papers to be part of this review. A complete view of the whole review process is shown in Figure 3.

## 3. Results

**Figure 4:**
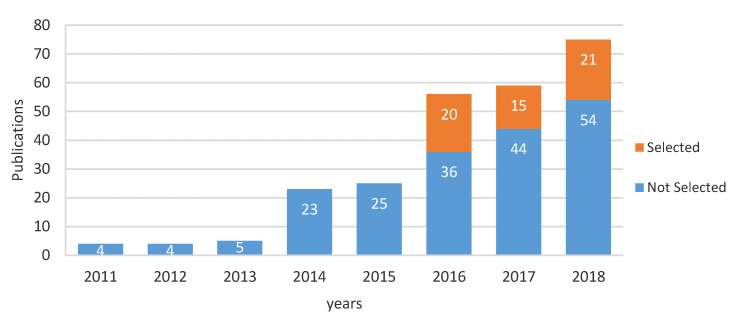
The trend in numbers of publications from papers initially selected for all years included in the database.

The main goal of this work is to gather information and provide knowledge about the hardware technologies used in activity recognition research for smart home and AAL as well as a road map for project development for companies or entrepreneurs who may want to get into this field. This section provides a significant overview of how hardware technology is being used. Activity recognition in smart home and ALL development of hardware is recent; the first documents gathered on the WoS database showed that publications in the field do not have even a decade, as shown in Figure 4. Due to the timing of journal publication, it is possible that hardware technology for activity recognition in smart homes and AAL started to be used more since 2010. There is no doubt that R&D in activity recognition for smart home and ALL is a trend that has increased year to year.

**Figure 5:**
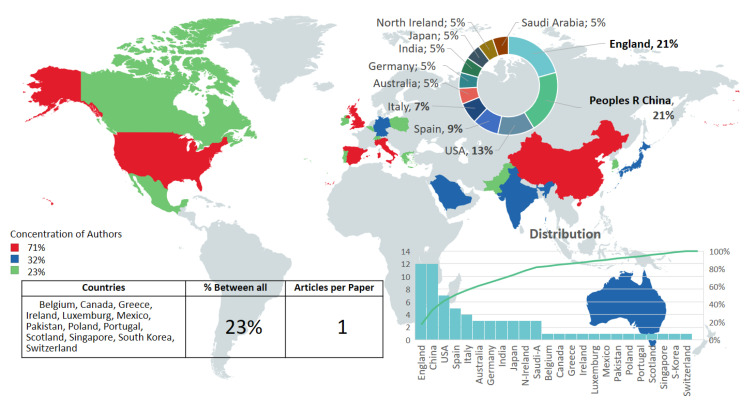
Worldwide map with an overview of the concentration and distribution of selected works.

From the selected papers, the WoS analysis tool shows that only 2.5% of countries published reports on deploying hardware in activity recognition for smart home and ambient assisted living. Of those, 71% of the authors were concentrated in England, China, USA, and Spain, and 32% of authors were in Australia, Germany, India, Japan, North Ireland, and Saudi Arabia. Only 23% of countries reported one author with one publication (Figure 5). Latin American and Africa did not appear in the analysis, which does not mean that these regions are not working in this field, but may be due to the focused database (WoS) used for the review. For example, in a study published by Latin American researchers [30], they use the Emotiv Insight wearable for emotion recognition to study emotional levels during task execution, applying a different data mining approach.

**Figure 6:**
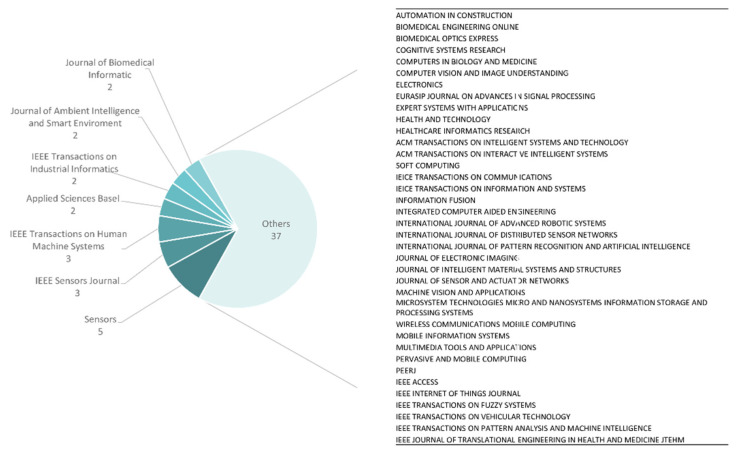
Overview of the journal distribution of selected papers.

There is no marked difference between the lowest and highest numbers of publications in journals. Despite that, we have to highlight that the Sensors Journal has the most publications, and IEEE, MDPI, IEIC, ACM, and Springer have a strong presence as publishers in this field of research. All journals with publications reported in this study are shown in Figure 6.

**Figure 7:**
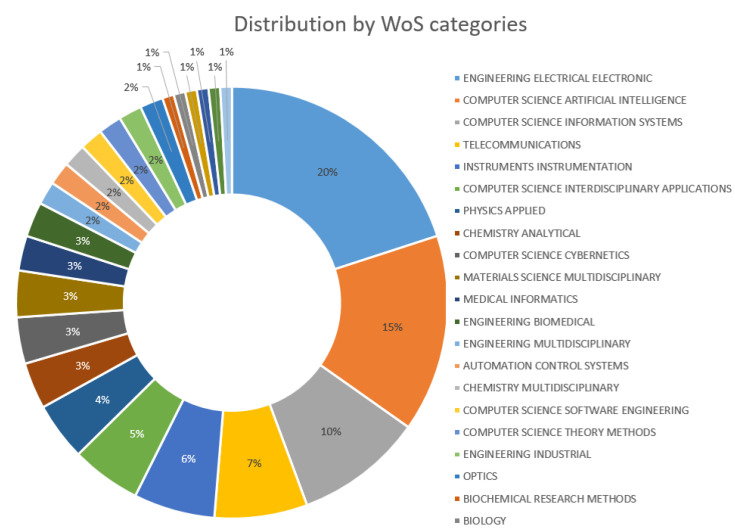
WoS research area distribution of the selected works.

The indexed categorization of WoS research areas has a marked fact (Figure 7), with engineering and computer science as the main areas of published works, followed by telecommunication and instrumentation categories. This is consistent with the type of hardware and software technology used to achieve the goals of activity recognition in smart home and AAL, as these are at the heart of the technology. Figure 7 also allows appreciating other research areas from which these hardware developments in AR for smart home and AAL are also carried out, such as physic, chemistry, biochemical, biology, medical, among others.

**Figure 8:**
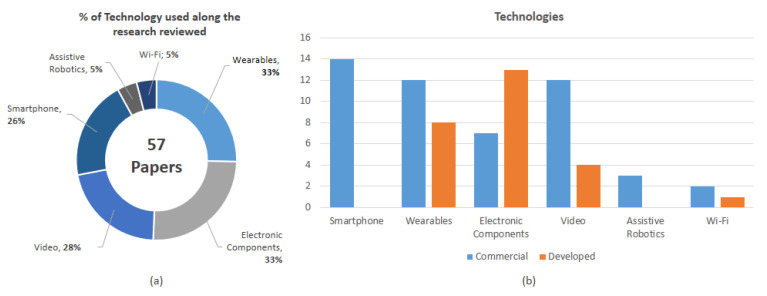
Analysis of hardware technology distribution. (**a**) Percentage of uses along the works reviewed. (**b**) Based on self-developed or commercial device-based solutions.

Smart home technology became a focus of the product market beyond a research topic [9]. This study found six groups of technologies; the four biggest are video, smartphone, wearable, and electronic components, and the other two are prominent in development: Wi-Fi and assistive robotics. Figure 8 shows the distribution of these technologies, and whether they are self-developed hardware or commercial end-user hardware without modification already available on the market as a final product. It shows the most used technologies in the research works reviewed as well.

**Figure 9:**
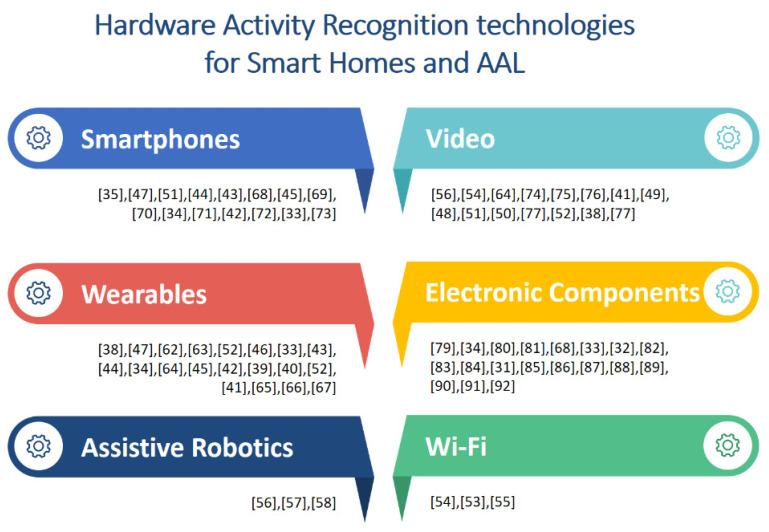
Identified categories of hardware technology used for activity recognition in smart home and ambient assisted living and its contributions.

Developing and prototyping hardware is an attractive alternative in activity recognition research for smart home and AAL, to build systems from scratch using kits, boards, or chipsets as Arduino, Seeeduino, Raspberry, low-power microcontroller (MCUs), and sensors which later require data acquisition units to process the data. Almost 50% of the studies use this type of hardware solution. On the other hand, 60% also use components based on “plug and play” devices and systems with low levels of configuration just for connecting and gathering data before process it, like wall-mounted PIR sensors [31], microphones [32], infrared cameras [33], active tags [34], and radio-frequency identification (RFID) systems [35]. We found some interesting developments around video solutions, not using regular video cameras as would be expected, but specialized video hardware. Many applications that use wearables are based on commercial smartwatches, but others are based on self-developed smart bands or commercial wearables sensor devices like Shimmer. Smartphone applications are used on commercial devices run on Android, iOS, and Windows Mobile. We put smartphones in a different category from wearables; even though we can hold them in our pockets, handbags, and hands, smartphones are not be worn on the body, as wristwatches, rings, glasses, and necklaces are, following the categorization of wearables defined in [36] as accessories, clothing, and on-skin. Despite close use of smartphones and wearables such as smartwatches in daily life nowadays, this review found that not all applications of wearables are based on integration with smartphones; many studies analyzed the use of electronic components as a built-in solution for creating one’s own wearables. Even so, these groups are just a broad categorization to facilitate an analysis of how this technology is being used together. It is worth highlighting that almost all studies had solutions using different technologies, so those are categorized into more than one group, as shown in Figure 9, showing a general view of the studies integrating different types of technology.

### 3.1. Wearables

New products like smart bands and smartwatches from big tech companies like Samsung, Apple, and Microsoft put on the map the concept of wearable technology. Wearable sensors are devices composed of tiny sensors that can be worn in an article of clothing or more unobtrusively, such as embedded rings, shirts, or watches, which gather body and context information to process or transmit it [6]. Wearable wireless sensor technology attracted social and corporate interest in areas such as enhancing independent living for disabled people, support for physical training and monitoring work, but even more in health care applications such as posture and movement recognition, real life-vision, rehabilitation systems, and respiratory and stress biofeedback assessment, among others [6]. The above may be due to emerging IoT technology and smart devices, sensors, and data processing hardware becoming commodities; on the other hand, the rising cost of healthcare systems induces wearable health tech research and new developments. Some wearable health devices are health regulatory authorized and successfully deployed, such as Nymy™, Samsung Gear™, and Apple Watch, not used for specialized or critical health issues but just to get biomedical signal data for daily life analysis [37]. We note commercial efforts in developing bendable smartphones, which can fall in the wearables zone. However, these are far from being used on the wrist due to the folded and flexible touchscreen display prototype level, besides that, none was found in this study.

A significant percentage of the papers based their experiments on self-developed technology or development tools for a wearable solution. Only 50% of the selected studies used commercial devices; others preferred to use modules, sensor boards, and related items. Accelerometers are a common factor among almost all of the studies, followed by gyroscopes. The rapid and low-cost accessibility, such as the flexibility of technology to build customized wearable combinations, allowed measuring variables in other parts of the body, such as heart rate in the chest [38]. On the other hand, interesting commercial wearable sensor bands like the Shimmer device are mentioned in more than one study [39,40,41].

The combination of wearables and smartphone technology is not apparent; only 37% of the studies used this combination, and just with specific smartwatch devices. Many wearables like smartwatches need to work with a smartphone, extending the functionality of the smartphone beyond data transmission, receiving and confirming text and audio messages, and taking and making calls. However, these smartwatches can work on their own for other purposes without being paired with a smartphone [7].

Mixing smartwatches with video capture and processing technology seems to be a field of work for various researchers. For the rest, it seems to be sufficient to use wearable technology alone to assess activity recognition for smart home and AAL, maybe to try simplicity in technological solutions. Commercial devices from big companies, such as Samsung Galaxy Gear Live [42], Microsoft Band 2 [43], and Intel Basis Peak [44], are mentioned in several studies, as well as other commercial alternatives like Empatica E3 [33], Fitbit [44], HiCling [34], Pebble [45], and Google Glass [33,46] (see Table 2).

### 3.2. Smartphones

Android seems to be a favorite platform to support activity recognition systems for smart home and AAL, not to say this is more effective than others, but this OS appears in most of the studies, except in [35,47], which used a smartphone but did not say which one, and [35], which used iOS. We did not identify any use of Windows Phone or any other mobile operative system. We did not identify a preferred model of Android phones. Besides, the use of wearable technology jumps out, and the elderly are the main benefiting population. Of the smartphone sensors, accelerometers are the most used, followed by GPS. Beyond generic AR applications for smart home and AAL, there is a focus on smartphones working in localization, occupancy, fall detection, posture recognition, and for the elderly population, disabled people, and health care (see Table 3).

### 3.3. Video

Activity recognition for smart home and AAL developed in video-based technology is popular. From the selected studies, 60% used RGB-D sensors, which are based mostly on the Kinect platform from Microsoft; only [48] uses an RGB camera from FLIR Systems. The authors of [49] combine RGB-D cameras with Vicon Systems cameras, and the authors of [48] use thermal cameras. Thermal cameras are used alone in [50] and with smartphones in [51]. There did not seem to be any interest in using video cameras combined with other technologies, more than with wearables [52] and infrared cameras [38] (see Table 4).

### 3.4. Electronic Components

Electronic components such as sensor boards, microcontrollers, board processors, electronic modules, communication devices, development toolkits, chipsets, and related devices, are mainly used to build from scratch or complement any function that a commercial device cannot provide. Electronic components appear in almost 30% of the selected research and they are one of the four main technologies used to build activity recognition for smart homes and AAL. Table 5 offers a complete overview of the types of hardware and some references, and models researchers worked with. Just a few works based on electronic components use other kinds of technology identified in this paper, such as [34], which uses active tags with smartphones and wearables, and [33], which uses a Raspberry board and an infrared camera taken from a Pupil Labs eye tracker and adapted for Google Glass. Electronic components are used for special activity recognition functions such as fall detection, localization, mobility, occupancy, posture recognition, and health, targeted to the elderly population.

### 3.5. Wi-Fi

The scientific community is concerned about nonintrusive activity recognition solutions. In this regard, this study presents an interesting way to apply AR for smart home and AAL: by using radio waves (Table 6). The above seems to be a promising solution by using a widely deployed technology, Wi-Fi routers. The authors of [53] captured information generated during radio wave propagation in indoor environments using wireless signals through a smart radio system that turns radio waves generated by Wi-Fi signals in an intelligent environment able to capture changes in multipath radio profiles, detecting motion and monitoring indoor events, even through walls in real time.

The authors of [54] present a human activity sense system for indoor environments called HuAc, based on a combination of Kinect and Wi-Fi. The system can detect even in conditions of occlusion, weak light, and activities with different perspectives such as forward kick, side kick, bending, walking, answering a phone, squatting, drinking water, and gestures like horizontal arm wave. In addition, this system also detects other activities such as two-handed waving, high throwing, tossing paper, drawing a tick mark, drawing an x, clapping hands, and high arm-waving.

The authors of [55] also use Wi-Fi links for evaluating passive occupancy inference problems. They set up signal processing methods and tools with electronic components to adapt this in a commercial Wi-Fi router. Based on the analysis of channel state information (CSI) collected from multiple-input-multiple-output (MIMO) using orthogonal frequency division multiplexing (OFDM) radio interfaces in off-the-shelf Wi-Fi networks, the system is capable of detecting localization of two independent human bodies moving arbitrarily through the working area of the system.

### 3.6. Assistive Robotics

**Figure 10:**
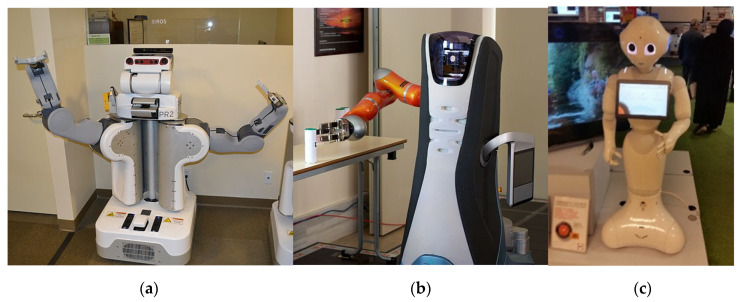
Assistive robots identified in activity recognition research: (**a**) PR2 robot [59]; (**b**) Pepper robot [60]; (**c**) Care-O-bot3 [61].

High technological level assistive robotics is used for developing applications on activity recognition for smart home and AAL, based on commercial robots and mainly focused on applications for health care and the elderly population. All studies use interactive robots manufactured in Germany, Japan, and the United States, as shown in Figure 10. Only the PR2 robot is being used in the same country [56], while Care-O-bot3 is used on collaboration between Portugal and Spain [57], and Pepper is used in the UK [58] (see Table 7).

The uses of PR2 [56] combine the robot with video capture through an RGB-D adapted to the robot’s head; with this camera, the robot can sense people’s movement. RGB-D sensors recognize people’s movements and anticipate future activity as a reactive response, called activity prediction. This is aimed at making smarter robots that can assist humans in making tasks more efficient or take on tasks that humans are unable to perform. Care-O-bot 3 is used in [57], in which AR is used to teach the robot to perform assisting tasks and behave in response to some tasks. The robot can identify some human activities thanks to the use of a fully sensorized system and ceiling-mounted cameras deployed in a house. The study mainly seeks to develop a robot personalization platform for end-users, as a robot system to teaching and learning for care workers and related helpers, and as a trusted companion for older adults as well. The above is a perfect example of how activity recognition systems can be matched with other technologies to achieve better living conditions.

PHAROS is a platform developed which uses the Pepper robot [58] to assist caregivers in teaching and evaluating the movements of adults in their daily physical activities. The PHAROS system identifies the elder person and, based on his physical condition, recommends a series of personalized and planned exercises. In a scheduled way, the robot is capable of capturing the attention of older adults, showing on the screen and describing by audio the exercises he should perform. Pepper’s camera provides the video input to recognize the activity and extract the skeletal data by Openpose software, which helps to label the activity being performed, and sends it to a module that registers the health status, and based on that, gives recommended exercises.

## 4. Analysis and Discussion

**Figure 11:**
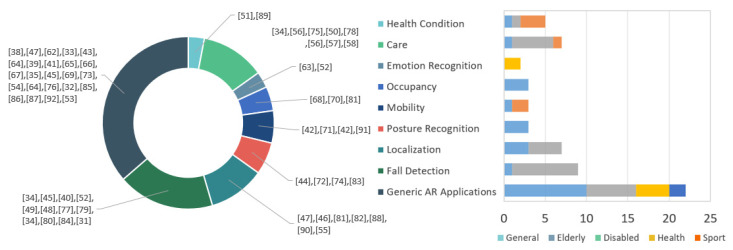
Distribution of activity recognition application in smart home and AAL and its relationship with target populations.

In the previous section, we described six main types of hardware technology used for activity recognition applied to the smart home and AAL research field. The majority of the reviewed works reported several goals of AR, with fall detection as the main one, followed by localization. Other AR applications were posture, mobility, occupancy, and emotion recognition. Many works did not report a specific goal, only a system capable of reaching it, or at least the authors of this review did not detect them, goals tagged as generic AR applications for smart home and AAL. Figure 11 shows an overview of how these goals are aimed at specific populations such as older adults through fall detection, localization, and care, and the disabled population through mobility, care, and health conditions. Surprisingly, emotion recognition seems to affect healthcare more than social or entertainment applications. Recognition of activities, events, and gestures is used to assess caregiving through behavioral patterns for health diagnostics. Generic AR applications refer to studies that did not mention a specific application or practical use.

**Figure 12:**
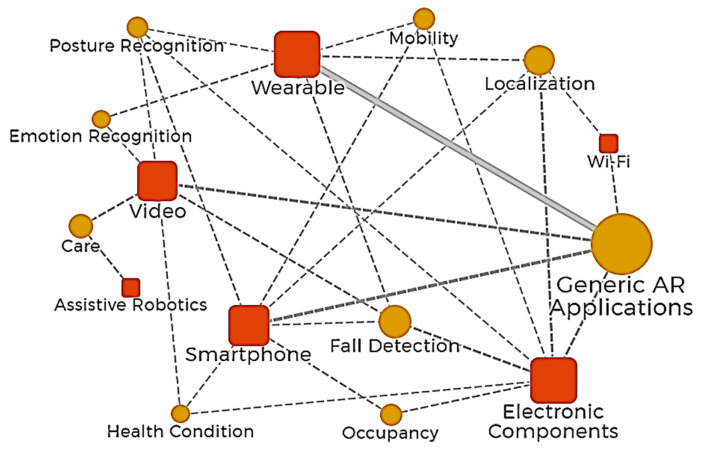
Relationships between technology (square) and research focus (circle) for activity recognition.

Results show specific relationships between types of technology and application focus of activity recognition for smart home and AAL. Figure 12 shows this relation through a relation network in which the size of the node means the frequency of technology use or application focus, and the thickness of the lines shows a greater or lesser relationship between both groups. Some reviewed works show applications such as occupancy based on technologies like electronic components and smartphones. In [68], the Android phone is used for data transmission through an app, with ultrasonic and passive infrared sensors, achieving height detection as a unique bio-feature, and efficient differentiation of multiple residents in a home environment. Other research also used electronic components and smartphones for medical treatment of health conditions, monitoring vital signs like respiratory rate. For example, [51] combined those technologies with video technology to achieve accurate respiratory rate tracking using an app phone for visualization and processing thermal images from a thermal camera (Flir One for Android).

For care applications, researchers combined video and assistive robot technology, using activity recognition as input for activity prediction to help the robot perform actions in response to human activity; a similar goal was achieved in [56], combining a PR2 robot with RGB-D sensor Kinect technology. Using only video technology can also help in elderly care; video helped estimate locations and perform behavioral analysis under low-resolution constraints as an alternative to PIR sensors or high-resolution cameras. For example, [78] used an Agilent ADNS-3060 optical sensor (30 × 30 pixels) installed in a service apartment for senior citizens, projecting pattern identification for recovery periods through caregiver monitoring.

Through video technology combined with wearables, some researchers project the use of emotion recognition applications such as monitoring and regulation emotions for patients in smart health environments, this is achieved by [52] using an electro-dermal activity (EDA) sensor with a low-power camera and Bluetooth data transmission. Fairly accurate recognition of emotions such as happy, neutral, and angry was achieved using only wearables, as done in [63], using the built-in accelerometer of a smart band. It is possible to achieve posture recognition using video, wearables, smartphones, and electronic components. An application like this could prevent decubitus ulcers through electronic components such as capacitive sensing, as the research work of [83], which a wired grid in a bedsheet with an OpenCapSense sensing unit, to help detect prolonged posture, allowing caregivers to be aware of this situation. Posture recognition using smartphones and wearables at the same time allows the mitigation of fake alarms in activity recognition. In [44], physiological sensors of smart bands like Fitbit and Intel Basis Peak are used to detect vital signs alarms; before the system sends an alarm, the user gives feedback about the situation through a screen and speech recognition mobile app, improving the accuracy of the activity recognition system and starting real-time communication with caregivers. Even for ambiguous posture detection, video technology is used for recognizing activities such as calling, drinking water, using a remote control, and pouring water.

Wearable, smartphone, and electronic component technologies also help to build solutions for activity recognition on mobile applications for smart home and AAL. In [42], a group of sensors such as accelerometer and heart rate sensors from a smartwatch, as well as a mic, accelerometer, GPS, and Wi-Fi traces from a smartphone was used to generate mobility pattern information from activities like walking, running, driving a vehicle, riding a bicycle, and inactive or sedentary activities.

Localization applications also use wearables and smartphones, achieving location-agnostic activity recognition. In [47], used customized sensor bands (accelerometer, rotation, and magnetic) placed on the arm and foot, using a smartphone as data transmission into the place, addressing home monitoring and privacy concerns for fine-grained lifelogging and cognitive prosthesis. Privacy concerns in localization use Wi-Fi commodity with some electronic components for passive occupancy inference [55], achieving detection and localization of two independent targets moving around the transmitter/receiver locations.

However, we point out from this relationship network analysis some interesting potential technological developments:

- Video technology can help in mobility and localization by using wearables as a way of alerting.
- Due to the prominent Wi-Fi results, research should extend to occupancy detection, fall detection, and posture for care.
- Assistive robots with wearables, smartphones, and electronic components can be used for vital sign monitoring and alerts for remote care.
- Wearables can be used for occupancy applications and care of health conditions.

**Figure 13:**
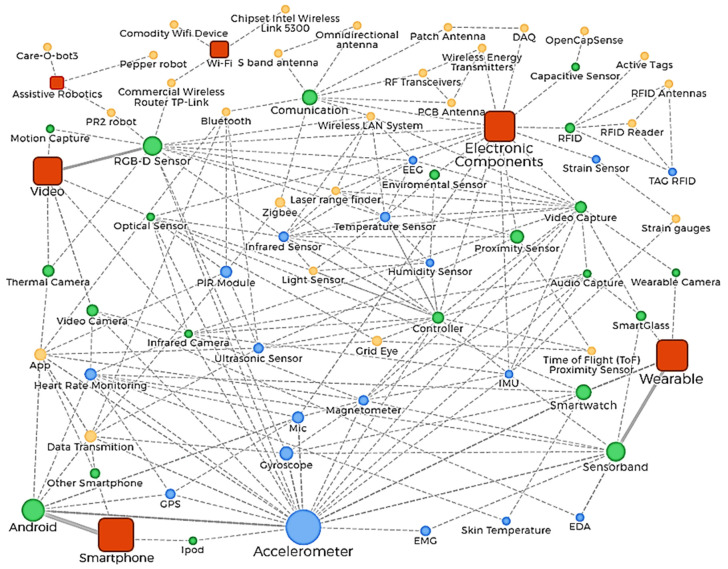
Relationship network analysis for hardware solutions deployed in activity recognition for smart home and AAL. Technology (orange square), a particular type of technology (green circle), itemized sensors (pink circle), and other specific devices (blue circle).

Some technologies that are less articulated with other technologies to develop solutions toward activity recognition for smart home and AAL, can be identified through a more in-depth relationship network analysis, as well as other points of interest stand out around the sensors or specific devices used for each technology identified in the present work (video, electronic components, wearables, smartphones, Wi-Fi, and assistive robots). The big panorama of deploying hardware technology for activity recognition for smart home and AAL shown in Figure 13, shows nodes with different colors representing the types of technologies, sensors, and devices. In this deeper relationship network, the size of each node represents the frequency of hardware use among the works reviewed, and the thickness of the lines between nodes represents how much these technologies are used in collaboration. This network relation uses a “has a” node hierarchy, like this: “Technology” has a “particular type of technology”, which has “sensors” and “other devices”. These last two are more detailed hardware info than the first two, which brings a better panorama about the hardware is being used in AR.

Video, electronic components, smartphones, and wearables show a trend of hardware used for AR in SH and AAL, these are the most frequently used among the technological solutions deployed; the relationship network (Figure 13) shows how these interact strongly through each type of technology. RGB-D sensors, video, and audio capture devices, infrared cameras, controller devices, optical sensors, wearable sensor bands, and smartwatches show amounts of collaborative solutions. Many papers included detailed information about sensors or devices used, highlighting strong collaborative solutions using apps for processing data, ultrasonic sensors, infrared and PIR modules, proximity sensors, temperature sensors, IMU, magnetometers, EEG, and heart rate monitoring. Other less strong, but still collaborative, are technologies like apps for data transmission, Bluetooth, Grid-EYE and time of flight, laser range finders, microphones, humidity sensors, and light sensors.

There is a potential roadmap for developing new solutions using technologies that are not currently being used very collaboratively with others, which researchers should study in future work, such as wearable cameras, strain gauges, skin temperature sensors, EDA sensors, smart glasses, GPS, electromyography (EMG) sensors, and Zigbee. Other technologies are far from joint solution deployment: assistive robots, Wi-Fi for passive detection, and capacitive sensors. Notice the novel technologies applied in activity recognition such as radiofrequency systems over S-band antennas, RF transceivers, antennas data acquisition systems, and RFID. The above may be due to the highly specialized knowledge needed to use and adapt these technologies for specific uses, more than data transmission. This last analysis shows that specific research goals in activity recognition cannot be achieved using one single hardware technology, but can through joint solutions. We consider essential try to integrate these technologies with others that are commonly used to expand the goal achievement of applications such as fall detection, localization, posture and occupancy recognition, care and health condition monitoring, and other potential applications.

Through this work, we identify how several hardware technologies are deployed for activity recognition around smart homes and AAL. We can now evaluate and determine which ones to develop and start to experiment from a secure starting point to address some societal issues, and to further close the knowledge gap in this field. This is the case of the Smart Home CUC laboratory starting in Colombia, for which this study will serve as raw information to plan the infrastructure, technology acquisition, and networking, and cross some research approaches (localization, mobility, etc.) with populations (elders, athletes, disabled, etc.) and local needs.

As this literature review was not planned to be deep but instead wide in coverage, it highlights some questions to be addressed in future works in order to give a broad and clear panorama of advances in technologies in this field, such as the following:

- How are large-scale house projects for activity recognition planned?
- Through technological surveillance, how can we extend our understanding of promising advances such as smart floors, smart beds, and smart walls?
- Which types of tested hardware technology are giving better results?
- How can researchers design testbeds? It is crucial to have an overview of how to design this type of experiment and increase the credibility for approval by scientific networks of new paper proposals.
- What is the cost-benefit relationship in achieving effectiveness in each focus of activity recognition?
- Which commercial technology gives the best effective results in activity recognition so that it can be taken to market?

All of these could open the door to new studies around activity recognition, helping reduce the time to market solutions.

## 5. Conclusions

This paper provides a detailed review of hardware technology used in activity recognition research related to smart homes and ambient assistive living (AAL) applications published in the last three years and indexed on the WoS database. The reviewed papers showed four main groups of hardware technology: smartphones, wearables, electronic components, and video. Half of the research approaches focus on fall detection, care, posture recognition, mobility, occupancy, emotion recognition, and health conditions. In contrast, the other half are not developed for any specific function, just for exploring and exploiting the available technology. RGB-D sensors and thermal and video cameras are the main video hardware to capture information. Android is the mobile operating system most used, usually with wearables and video technology. Two other technologies identified as emerging fields of study for applications in activity recognition in smart home and AAL are Wi-Fi and assistive robots. The first one has potential as a non-intrusive and invisible technology. Assistive robots are used to assist and guide human activity for health, and activity recognition is being implemented as a function of this type of robot.

From a relationship network analysis between types of technology and applications for activity recognition in smart homes and AAL, the review points out some interesting new potential developments combining some technologies. One of these is the use of video technology to help mobility and localization with wearables as a way of alerting. Another is to extend research to occupancy detection, fall detection, and posture for care due to the prominent Wi-Fi results. Another new solution is to use assistive robots with wearables, smartphones, and electronic components for vital sign monitoring and alerts for remote care, also the use of wearables for occupancy and care of health conditions.

Through a more in-depth relationship analysis of hardware uses in terms of sensors or specific devices used in each technology identified, the review also detected some lack of articulation of developing solutions toward activity recognition: wearable cameras, strain gauges, skin temperature sensors, EDA sensors, smart glasses, GPS, EMG sensors, and Zigbee. Others far from joint solution deployment are assistive robots and Wi-Fi for passive detection with technologies such as capacitive sensors, S-band antennas, RF transceivers, antenna data acquisition systems, and RFID. Assistive robots and Wi-Fi can be combined with others commonly used to expand the spectrum of applications for activity recognition in smart homes and AAL, with devices such as RGB-D sensors, video, and audio capture devices, infrared cameras, controller devices, optical sensors, wearable sensor bands, smartwatches, Android phones, apps for processing data, ultrasonic sensors, infrared and PIR modules, proximity sensors, temperature sensors, IMU, magnetometers, EEG, and heart rate monitors.

Further research could also expand and update the notion about hardware uses for activity recognition, for instance in other sources like Scopus, Google Scholar, or patent databases, as part of technological surveillance for monitoring these advances, and to study the effectiveness of these developments and find novel combinations and promising hardware that can help accelerate innovations in activity recognition field for smart home and ambient assisted living.

## Figures and Tables

**Table 1:** Transformation of common terms detected in the bibliometric networks analysis.

Interest Area	Common Term from VOSviewer	Duplication Frequency	Chosen Terms	Primary Query Strings
Ambient assisted living (AAL)	ALL	11	AAL Ambient assisted Assistance Assistive	**AAL query:** AAL OR “ambient assisted” OR assistance OR assistive
	Ambient assisted	11		
	Assisted	4		
	Ambient	4		
	Ambient assisted living	3		
	Assisted technology	2		
	ALL platform	1		
	ALL service	1		
	ALL system	1		
Smart home (SH)	Smart home	9	Smart home Environment Device House	**SH query:** Smart AND (home OR environment OR house OR device) OR intelligence
	Smart home technology	6		
	Smart home system	5		
	Smart home device	3		
	Smart house	1		
	Smart device	1		
Smart environment (SE)	Smart environment	6	Smart, environment, intelligence, home	
	Home environment	3		
	Intelligent environment	2		
	Smart environment	1		
	Intelligence	1		
Activity recognition (AR)	Activity	18	Activity Recognition “Human activity” “Human action” “Event detection” Action	**AR query:** Activity OR recognition OR “human activity” OR action OR “human action” OR “event detection”
	Recognition	14		
	Human activity	7		
	Human activity recognition	4		
	Activity recognition system	3		
	Action recognition	2		
	Human action recognition	2		
	Recognition system	1		
	Human action	1		

**Table 2:** Characterization of wearable technology used in selected papers.

Wearable Technology Used				Context of the Proposal			AR Solution		
Model	Type	Sensor	Body Part	Combination	Applications	Target	Commercial	Developed	Ref.
Customized	Wearable sensor band	Accelerometer + heart rate sensor	Chest + limb	Video	Generic AR applications	All		X	[38]
Customized		Accelerometer + Gyroscope + Magnetometer	Arms	Smartphone	Generic AR applications + Localization	Elderly		X	[47]
Customized		Accelerometer + Gyroscope	Arm	-	Generic AR applications	Health		X	[62]
Customized		Accelerometer	Hand	-	Emotion recognition	Health		X	[63]
Customized	Skin sensor	Electro-dermal activity (EDA)	Skin	Video	Emotion recognition	Health		X	[52]
Google Glass Explorer	SmartGlass	Video capture	Head	-	Localization	Elderly	X		[46]
Google Glass-based + Head tracking device + Empatica E3 sensor armband	SmartGlass + smart band	IMU + Audio + Video	Head + Arm	Electronic components + Smartphone + Video	Generic AR applications	Elderly	X	X	[33]
Microsoft Band 2	Smartwatch	Accelerometer	Arms	Smartphone	Generic AR applications	All	X		[43]
Fitbit + Intel Basis Peak		Heart rate monitoring + Skin temperature monitoring	Hand	Smartphone	Posture recognition	All	X		[44]
HiCling		Optical sensor + Accelerometer + Captive skin touch sensor	Arms	Electronic Components + Smartphone	Fall detection	All	X		[34]
NS		Accelerometer + Gyroscope	Arms	Video	Generic AR applications	All	X		[64]
Pebble SmartWatch		3-axis integer accelerometer	Arms	Smartphone	Fall detection	Elderly	X		[45]
Samsung Galaxy Gear Live		Accelerometer + Heart rate sensor	Arms	Smartphone	Mobility	All	X		[42]
Shimmer	Wearable sensor band	Accelerometer	Wrist	-	Generic AR applications	Elderly	X		[39]
Shimmer		Accelerometer + Gyroscope	Abs	-	Fall detection	Elderly	X		[40,52]
Shimmer		Accelerometer + Gyroscope	Wrist	Video	Generic AR applications	Elderly	X		[41]
WiSE		Accelerometer	Arms	-	Generic AR applications	Sport		X	[65]
WiSE		Electrodes + Accelerometer	Arms	-	Generic AR applications	Sport		X	[66]
Microsoft Sens Cam	Wearable camera	Video capture	Chest	-	Generic AR applications	All	X		[67]

**Table 3:** Characterization of smartphone technology used in selected papers.

Smartphone Uses		Context of the Proposal			AR Solution		
Model	Sensor Applied	Combination	Applications	Target	Commercial	Developed	
Smartphone	Data transmission	-	Generic AR applications	Elderly	X		[35]
Smartphone	Data transmission + App	Wearable	Generic AR applications + Localization	Elderly		X	[47]
Android	App	Video	Health conditions	All	X		[51]
Android	Accelerometer	Wearable	Posture recognition	All	X		[44]
Android	Mic	Wearable	Generic AR applications	All	X		[43]
Android	Data transmission + App	Electronic Components	Occupancy	All		X	[68]
Android	Data transmission + App	Wearable	Generic AR applications	Elderly	X		[45]
Google NEXUS 4	Accelerometer	-	Generic AR applications	Health	X		[69]
Google NEXUS 5	Accelerometer + Mic + Magnetometer	-	Occupancy	All	X		[70]
HTC802w	Accelerometer + GPS	Electronic Components + Wearable	Fall detection	All	X		[34]
IPod Touch	Accelerometer	-	Mobility	Disabled	X		[71]
LG Nexus 5	Accelerometer + Mic + GPS + Wi-Fi	Wearable	Mobility	All	X		[42]
Samsung ATIV	Accelerometer Gyroscope	-	Posture recognition	All	X		[72]
Samsung Galaxy S4	Accelerometer + Mic	Electronic Components + Wearable + Video	Generic AR applications	Elderly	X	X	[33]
Xolo era 2x and Samsung GT57562	Accelerometer	-	Generic AR applications	All	X		[73]

**Table 4:** Characterization of video technology used in selected papers.

Video Technology		Context of the Proposal			AR Solution		
Type	Model	Combination	Applications	Target	Commercial	Developed	Ref.
RGB-D Sensor	Kinect	Assistive robotics	Care	All	X		[56]
	Kinect	Wi-Fi	Generic AR applications	All	X		[54]
	Kinect	Wearable	Generic AR applications	All	X		[64]
	Kinect	-	Posture recognition	All	X		[74]
	Kinect	-	Care	Disabled + Elderly	X		[75]
	Kinect	-	Generic AR applications	All	X		[76]
	Kinect	Wearable	Generic AR applications	Elderly	X		[41]
RGB-D Sensor + Vicon System camera	Kinect + Vicon System camera	-	Fall detection	Elderly	X		[49]
RGB-D sensor + Thermal camera	Thermal camera PI450 Grasshopper RGB GS3-U3-28S5C-C FLIR	-	Fall detection	Elderly		X	[48]
Thermal camera	FLIR One for Android	Smartphone	Health conditions	All	X		[51]
	FLIR E60 thermal infrared camera	-	Care	Elderly	X		[50]
Video camera	-	-	Fall detection	Elderly	X		[77]
	-	Wearable	Emotion recognition	Health		X	[52]
Video camera + Infrared camera	-	Wearable	Generic AR applications	All		X	[38]
Optical sensor	Agilent ADNS-3060 Optical mouse sensors	-	Care	Elderly		X	[78]

**Table 5:** Characterization of electronic component technology used in selected papers.

Electronic Components Used		Context of the Proposal			AR Solution		
Technologies	Reference/Model	Combination	Applications	Target	Commercial	Developed	Ref.
TAG RFID + RFID antennas + RFID reader	Smartrack FROG 3D RFID + RFID reader antennas + Impinj Speedway R-420 RFID reader	-	Fall detection	Elderly	X		[79]
Active tags	-	Smartphone + Wearable	Fall detection	All	X		[34]
Grid-EYE + Ultrasonic sensor + Arduino	Grid-EYE (AMG8853, Panasonic Inc.) hotspot detection + Ultrasonic HC-SR04 + Arduino Mega	-	Fall detection	Elderly		X	[80]
Gird-EYE + Rotational platform + Time of flight (ToF) ranging sensor + Arduino	Gird-EYE AMG 8853 Panasonic VL53L0X + Arduino Nano	-	Localization + Occupancy	All		X	[81]
HC-SR04 + PIR module + BLE module	-	-	Occupancy	All	X		[68]
Infrared camera Raspberry	Pupil Labs eye tracker Raspberry Pi 2	Smartphone + Wearable	Generic AR applications	Elderly	X	X	[33]
Microphone	-	-	Generic AR applications	All	X		[32]
Zigbee transceiver ultra-low-power microcontroller	CC2520 + MSP430F5438 chipsets.	-	Localization	All		X	[82]
Capacitive sensing	OpenCapSense sensing toolkit	-	Posture recognition	Health		X	[83]
XBee Pro + Series Pro 2B antennas + Laser diode	Part 2 XBee Pro + Series Pro 2B antennas + NR	-	Fall detection	Elderly		X	[84]
PIR sensors	-	-	Fall detection	Elderly	X		[31]
PIR sensor + Motion sensor + Data sharing device	NR sensor + PogoPlug		Generic AR applications	All	X		[85]
S-band antenna + Omnidirectional	-	-	Generic AR applications	Health	X		[86]
Seeeduino + Temperature and humidity sensor + Light sensor + Ranging sensor + Microphone	Seeeduino Arch-Pro + HTU21D + Avago ADPS-9960 + GP2Y0A60SZ + Breakout board INMP401	-	Generic AR applications	All		X	[87]
Sensor node consisting of nine PIR sensors arranged in a grid shape + CC2530 Zigbee module	CC2530 used to sample PIR signals and communicate with the sink node	-	Localization	Elderly		X	[88]
Strain gauge sensor + IMU sensor	SGT-1A/1000-TY13 StrainGauges + LSM9DS1 9axis IMU	-	Health conditions	Elderly		X	[89]
Measurement setup: low-noise amplifier (LNA), data-acquisition unit (DAQ) + Switching SP64T + Downconverter unit + Path antennas		-	Localization	Elderly		X	[90]
Portable brain-activity measuring equipment NIRS-EEG probes and NIRS-EEG unit + Thermometer + Laser range finder + Kinect + Pyroelectric sensor + Wireless LAN system + Sensor arrangement cameras + Microphones + Infrared devices		-	Mobility	Disabled		X	[91]
Tunable RF transceivers NI USRP-2920 + MIMO cable Wireless energy transmitter + PCB antennas		-	Generic AR applications	Health		X	[92]

**Table 6:** Wi-Fi devices used as the main component of activity recognition.

Wi-Fi uses		Context of the Proposal		AR Solution		
Technology	Reference	Combination	Applications	Commercial	Developed	Ref.
Wireless router	Commercial TP link	Video	Generic AR applications	X		[54]
Commercial Wi-Fi device	NS	-	Generic AR applications	X		[53]
Wi-Fi + Chipset	NS	Electronic components	Localization		X	[55]

**Table 7:** Characterization of assistive robotics used in selected papers.

Assistive Robotics Technology for AR					
Technology	Combination	Goal	Target	AR Solution	Ref.
PR2 robot	Video + RGB-D sensor	Care	All	Commercial	[56]
Care-O-bot3	-	Care	Elder	Commercial	[57]
Pepper robot	-	Care	Elder	Commercial	[58]
